# Identification of *C16orf74* as a Marker of Progression in Primary Non-Muscle Invasive Bladder Cancer

**DOI:** 10.1371/journal.pone.0015260

**Published:** 2010-12-21

**Authors:** Won Tae Kim, Seok Joong Yun, Cheol Park, Isaac Yi Kim, Sung-Kwon Moon, Tae Gyun Kwon, Yung Hyun Choi, Wun-Jae Kim

**Affiliations:** 1 Department of Urology, College of Medicine, Chungbuk National University, Cheongju, Republic of Korea; 2 Section of Urologic Oncology, The Cancer Institute of New Jersey, Robert Wood Johnson Medical School, New Brunswick, New Jersey, United States of America; 3 Department of Food and Biotechnology, Chungju National University, Chungju, Republic of Korea; 4 Department of Urology, School of Medicine, Kyungpook National University, Daegu, Republic of Korea; 5 Department of Biomaterial Control, Dong-Eui University, Busan, Republic of Korea; Florida International University, United States of America

## Abstract

**Purpose:**

Methylation-induced silencing of *PRSS3* has been shown to be significantly associated with invasive bladder cancer, and expression of the *C16orf74* gene locus has been shown to correlate positively with *PRSS3.* The aim of the current study was to evaluate the relationship between *C16orf74* expression level and progression in non-muscle invasive bladder cancer (NMIBC).

**Materials and Methods:**

*C16orf74* mRNA levels were examined by real-time reverse transcriptase polymerase chain reaction (RT-PCR) analysis of 193 tumor specimens from patients with primary NMIBC. Expression data were analyzed in terms of clinical and experimental parameters. Kaplan-Meier curves and multivariate Cox regression models, respectively, were used to determine progression-free survival and to identify independent predictive parameters of progression.

**Results:**

Analysis using Kaplan-Meier curves revealed prolonged progression-free survival of high-*C16orf74*-expressors as compared to low-expressors (p<0.001). Multivariate Cox regression analysis revealed that low *C16orf74* mRNA expression levels are a significant risk factor for disease progression in patients with primary NMIBC (HR: 10.042, CI:2.699–37.360, p = 0.001).

**Conclusions:**

Decreased expression of *C16orf74* correlates significantly with progression in primary NMIBC. *C16orf74* expression level represents a potentially useful marker for predicting progression in primary NMIBC patients.

## Introduction

More than 90% of bladder cancers are transitional cell carcinomas, and most are papillary, well-, or moderately-differentiated non-muscle invasive bladder cancer (NMIBC) [1]–[2]. After endoscopic resection, cancer recurrence occurs in the majority (50–70%) of patients with NMIBC [3]. Approximately 20% of these patients subsequently experience disease progression to muscle invasive bladder cancer (MIBC) after appropriate treatment, including transurethral resection (TUR) and intravesical therapy with epirubicin, mitomycin-C, or Bacillus Calmette-Guerin (BCG) [1]–[2]. Thus, frequent recurrence after TUR and subsequent cancer progression are problematic for patients and urologists alike. Almost 25% of newly diagnosed bladder cancer patients have MIBC, and the vast majority of these cases are of high histological grade. Nearly 50% of patients with MIBC already have occult distant metastases at the time of diagnosis [1]–[2].

A number of potential tumor markers have been identified for bladder cancer, but few have demonstrated efficacy in terms of predicting disease recurrence and progression. However, several recent studies have suggested that the suppressor genes *p53*, *RUNX3*, *RASSF1A*, and *PRSS3* are closely associated with the development and progression of bladder cancer [4]–[7]. Specifically, *RASSF1A* and *PRSS3* promoter methylation is associated with advanced tumor stage [7], which suggests that these genes might be associated with bladder cancer progression. *PRSS3* in turn has been shown to be positively associated with *C16orf74* expression [8].

The *C16orf74* (MGC17624) gene locus is on chromosome 16q24.1, and its function has yet to be characterized. The results of several genome-wide studies have indicated that *C16orf74* is involved in inflammatory processes. Tumor necrosis factor (TNF)-α is a key regulator of the inflammatory cascade in chronic inflammatory diseases, and in patients with inflammatory disease, *C16orf74* is strongly associated with an anti-TNF response [9]. *C16orf74* is a hypoxia regulated gene [10]–[11]. Winter et al. [10] reported that *C16orf74* median RNA expression level is an independent prognostic factor for recurrence-free survival in head and neck cancer. *C16orf74* has also been shown to be upregulated in lymph node-positive metastases in patients with oral tongue squamous cell carcinoma [12], and to correlate positively with *PRSS3* expression in breast cancer [8].

Recently, we reported the identification of a progression-related gene classifier that had strong predictive value in terms of disease outcomes in NMIBC [13]. In that study, *C16orf74* was one of eight candidate genes identified for predicting disease progression in NMIBC, suggesting a potential relationship between bladder cancer and *C16orf74*. In the current study, we assessed the relationship between *C16orf74* and NMIBC outcomes using data from a previous study population as well as new cases, all with long-term follow-up.

## Results

### 1. Baseline characteristics

The mean age of the 193 subjects with primary NMIBC was 64.1±14.0 years, and the median follow-up period was 44.9 months. Seventy-one patients (36.8%) experienced recurrence and 20 (10.4%) experienced progression. Other baseline characteristics of the patients are presented in Table 1.

**Table 1:** Baseline characteristics of primary non-muscle invasive bladder cancer patients.

Variables	Incidence or mean value (%)
Age (years)	64.1±14.0
Median follow up periods (months)	44.9
Gender	
Male	157 (81.3)
Female	36 (18.7)
Size	
<3 cm	109 (56.5)
≥3 cm	84 (43.5)
Number	
Single	111 (57.5)
Multiple	82 (42.5)
Grade	
G1	67 (34.7)
G2	101 (52.3)
G3	25 (13.0)
Stage	
Ta	71 (36.8)
T1	122 (63.2)
Intravesical therapy	
No	80 (41.5)
Yes	113 (58.5)
Recurrence	
No	122 (63.2)
Yes	71 (36.8)
Progression	
No	173 (89.6)
Yes	20 (10.4)

### 2. The value of *C16orf74* mRNA expression level as a prognostic marker for progression

**Figure 1:**
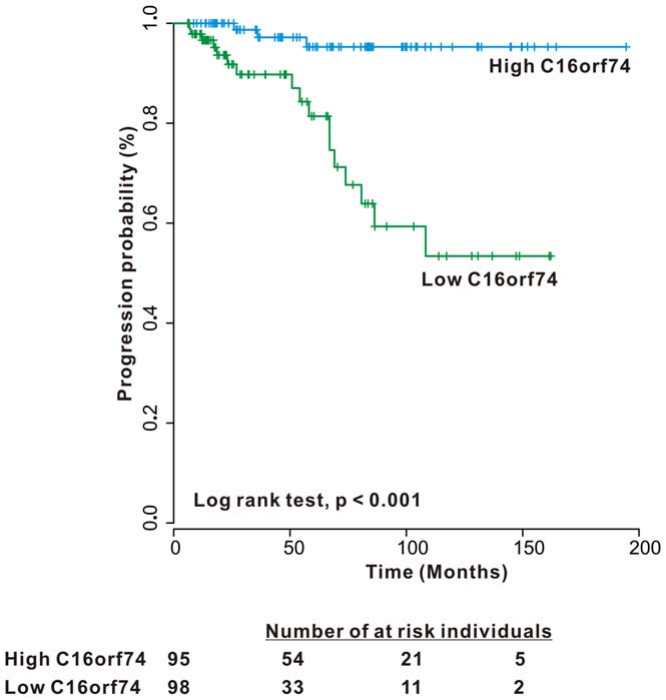
Time to progression in non-muscle invasive bladder cancer according to *C16orf74* mRNA expression levels.

The relationship between *C16orf74* mRNA expression level and time to progression was analyzed. Using a ROC curve, a cutoff value (11.7784) for progression with the highest combined sensitivity (53.2%) and specificity (85%) was determined. Time to progression was significantly different between the high and low *C16orf74* mRNA expression groups, in that time to progression in the high *C16orf74* expression group was significantly longer than the low expression group (p<0.001) (Fig. 1). In univariate Cox regression analysis of several clinicopathological variables (age, sex, tumor size, number, grade, stage, intravesical therapy, and *C16orf74* mRNA expression levels), age, intravesical therapy and *C16orf74* mRNA expression levels were significant risk factors for progression (p = 0.031, p = 0.034 and p<0.001, respectively). In multivariate Cox regression analysis, age and low *C16orf74* mRNA expression levels were significant risk factors for progression-free survival in patients with primary NMIBC (HR: 1.049, CI: 1.005–1.094, p = 0.030; and HR: 10.042, CI: 2.699–37.360, p = 0.001, respectively) (Table 2). In multivariate Cox regression analysis in patients with intravesical therapy, age and low C16orf74 mRNA expression levels were significant risk factors for progression-free survival in patients with primary NMIBC with intravesical therapy (HR1.055, CI: 1.005–1.108, p = 0.031; and HR: 14.170, CI: 2.719–73.837, p = 0.002, respectively).

**Table 2:** Multivariate Cox regression analysis for prediction of progression in NMIBC and in NMIBC with intravesical therapy.

Variables	Total Patients (N = 193)				Intravesical Tx. Pt. (N = 113)	
	Univariate Cox regression		Multivariate Cox regression		Multivariate Cox regression	
	HR (95% CI)	p-value	HR (95% CI)	p-value	HR (95% CI)	p-value
Age (years)	1.043 (1.004–1.083)	0.031	1.049 (1.005–1.094)	0.030	1.055 (1.005–1.108)	0.031
Sex (male vs. female)	0.627 (0.145–2.706)	0.531	0.398 (0.078–2.035)	0.268	0.780 (0.140–4.352)	0.777
Size (<3 cm vs. ≥3 cm)	1.912 (0.781–4.680)	0.156	2.222 (0.808–6.109)	0.122	1.801 (0.560–5.793)	0.324
Number (Single vs. multiple)	1.885 (0.779–4.562)	0.160	1.391 (0.522–3.706)	0.509	1.424 (0.456–4.452)	0.543
Grade	2.076 (0.989–4.360)	0.054				0.586
G1	1	-	1	-	1	-
G2	2.143 (0.698–6.574)	0.183	0.601 (0.130–2.785)	0.515	0.337 (0.042–2.683)	0.304
G3	4.279 (0.942–19.428)	0.060	0.735 (0.090–5.998)	0.774	0.317 (0.024–4.269)	0.386
Stage (Ta vs. T1)	1.294 (0.470–3.565)	0.618	0.608 (0.168–2.195)	0.447	0.865 (0.145–5.151)	0.874
Intravesical Tx. (No vs. Yes)	3.765 (1.102–12.863)	0.034	2.840 (0.666–12.109)	0.158	-	-
C16orf74 (high vs. low)	8.940 (2.614–30.576)	<0.001	10.042 (2.699–37.360)	0.001	14.170 (2.719–73.837)	0.002

Tx.: Therapy, Pt.: patients, HR: hazards ratio, CI: confidence interval.

## Discussion

Trypsin is a member of the serine protease family encoded by three trypsinogen genes including *PRSS1*, *PRSS2* and *PRSS3* encode trypsinogen I, trypsionogen II, and trypsinogen IV (also known as mesotrypsinogen), respectively [14]–[16]. This enzyme has been known as a potent proteolytic enzyme that can destroy tissue [17]–[18]. There are conflicting reports in the literature of the role of trypsin or PRSS3 in tumor progression, with some studies assigning a positive role [19]–[23], while others have reported that trypsin or PRSS3 plays a tumor suppressive role. The expression of PRSS3 is reduced in bladder, esophageal, and gastric cancers, and loss of PRSS3 expression is due to epigenetic silencing through promoter hypermethylation [7], [24]–[25]. In particular, silencing of *PRSS3* by promoter methylation has been significantly associated with invasive tumor stage in bladder cancer [7].

The expression of *C16orf74* has been shown to correlate positively with *PRSS3*. Hockla et al. [8] reported that *C16orf74* is down regulated by knockdown of *PRSS3* and upregulated by mesotrypsin treatment. To date, there have been no reports of an association of *C16orf74* with bladder cancer, except as indicated in an earlier work by the authors [13]. Here, we analyzed the relationship between mRNA expression levels of *C16orf74* and progression in primary NMIBC. Reduced expression of *C16orf74* was significantly associated with disease progression in NMIBC patients, suggesting that *C16orf74* has a tumor suppressive role, similar to *p53, RUNX3* and *PRSS3*, in disease progression. To date, the function of *C16orf74* is unknown, and additional studies are needed to define the precise pathway by which *C16orf74* influences progression in primary NMIBC.

Generally, clinical and pathological parameters such as tumor grade, tumor stage, lymphatic invasion, tumor size, CIS, papillary or solid tumor architecture, and multifocality have been considered useful prognostic parameters for disease progression in NMIBC. Of these factors, generally tumor grade, stage, and presence of CIS are considered the most important. In the current study, intravesical therapy was a risk factor for progression upon univariate analysis. However, it is possible that patients who received intravesical therapy were in a clinically high risk group for recurrence or progression, rather than that the treatment affected progression [26]. Various molecular markers have also been evaluated for disease progression. Recently, several studies have identified putative progression-related genes in NMIBC using gene expression analysis [27]–[29]. Wang et al. [27] proposed a 57-gene panel to help predict progression in NMIBC. Birkhahn et al. [28] reported that *HRAS*, *VEGFR3*, and *VEGF* expression levels were related to progression with 81% sensitivity and 94% specificity. Eguchi et al. [29] reported that the loss of 8p23.3 is a marker for predicting progression and recurrence in NMIBC. Previously, we identified a candidate progression-related gene classifier that had strong predictive value in terms of disease outcomes in NMIBC [13]. Although *C16orf74* is a single molecular marker within this candidate progression-related gene classifier, it was sufficient to predict the risk of progression in NMIBC with a strong hazard ratio of more than 10 upon multivariate analysis.

In the current study, we investigated the mRNA expression levels of *C16orf74* in human primary NMIBC tissues in a relatively large population with a long-term follow up period, along with several known clinical risk factors, including age, tumor size, number of tumors, T-category, tumor grade, and intravesical therapy [30]–[31]. These aspects of the study design lend strength to the results, and strongly suggest that *C16orf74* may be a clinically useful predictor of progression in primary NMIBC.

In conclusion, decreased expression of *C16orf74* was significantly associated with progression in primary NMIBC, and the expression level of *C16orf74* was an independent prognostic determinant for tumor progression. *C16orf74* might play a key role in the progression of NMIBC. Thus, *C16orf74* expression level represents a useful marker for predicting progression in primary NMIBC patients.

## Materials and Methods

### 1. Ethics Statement

The Ethics Committee of Chungbuk National University approved this protocol, and written informed consent was obtained from each subject. Collection and analysis of all samples was approved by the Institutional Review Board of Chungbuk National University.

### 2. Patients and Tissue Samples

Primary NMIBC samples from patients with histologically-verified transitional cell carcinoma obtained at our institute were used for the current study. Patients with concomitant carcinoma *in situ* (CIS) or a short term follow-up period (less than 6 months), and those that underwent radical cystectomy or for whom there was incomplete data collection, were excluded to make the study population more homogeneous. A total of 193 primary NMIBC samples were analyzed.

All tumors were macrodissected, typically within 15 minutes of surgical resection. Each bladder cancer specimen was confirmed by pathological analysis of a part of the tissue sample in fresh frozen sections from TUR specimens, and was then frozen in liquid nitrogen and stored at −80°C until use. A second TUR was performed 2–4 weeks after the initial resection when a bladder cancer specimen did not include proper muscle or when high-grade tumor was detected [32]. Patients who had a T1 tumor, multiple tumors, large tumors (≥3 cm in diameter), or high grade Ta NMIBC received one cycle of intravesical treatment (BCG or mitomycin-C) [26], [32]. If a patient refused intravesical therapy, it was not administered after TUR. Response to treatment was assessed by cystoscopy and urinary cytology. Patients who were free of disease within 3 months after treatment were assessed every 3 months for the first 2 years and then every 6 months thereafter [26], [32]. Tumors were staged and graded according to the 2002 TNM classification and the 1973 WHO grading system, respectively [32]–[33]. Recurrence was defined as recurrence of primary NMIBC with a lower or the same pathological stage, and progression was defined as disease with a higher TNM stage upon relapse.

### 3. RNA extraction and construction of cDNA

RNA was isolated from tissue using 1 ml of TRIzol (Invitrogen, Carlsbad, CA) and homogenization in a 5-ml glass tube. The homogenate was transferred to a 1.5-ml tube and then mixed with 200 µl of chloroform. After incubation for 5 min at 4°C, the homogenate was centrifuged for 13 minutes (min) at 13,000×*g* at 4°C. The upper aqueous phase was transferred to a clean tube and then 500 µl of isopropanol were added. The mixture was incubated for 60 min at 4°C, and then the tube was subjected to centrifugation for 8 min at 13,000×*g*, 4°C. The upper aqueous phase was discarded and mixed with 500 µl of 75% ethanol, and then centrifuged for 5 min at 13,000×*g*, 4°C. After discarding the upper aqueous layer, the pellet was dried at room temperature, dissolved in diethylpyrocarbonate (DEPC)-treated water, and then stored at −80°C. The quality and integrity of the RNA were confirmed by agarose gel electrophoresis and ethidium bromide staining, followed by visual inspection under ultraviolet light. cDNA was prepared from 1 µg of total RNA using a First-Strand cDNA Synthesis Kit (Amersham Biosciences Europe GmbH, Freiburg, Germany) according to the manufacturer's protocol.

### 4. Real-time PCR

Real-time PCR amplification was performed using a Rotor Gene 6000 instrument (Corbett Research, Mortlake, Australia) to quantify the expression of *C16orf74*. Real-time PCR assays were carried out in micro-reaction tubes (Corbett Research, Mortlake, Australia) using SYBR Premix EX Taq (TAKARA BIO INC., Otsu, Japan). The primers used for amplification were *C16orf74* (179 basepairs) sense (5′-AAT GTG TGT CAG CAG CAG CA-3′) and anti-sense (5′-TTT CTC CAT CAT CTG GGC AC-3′). The PCR reaction was performed in a final volume of 10 µl consisting of 5 µl of 2 X SYBR premix EX Taq buffer, 0.5 µl each of 5′- and 3′- primer (10 pmol/µl), and 1 µl of the sample cDNA. The product was purified with a QIAquick Extraction kit (QIAGEN, Hilden, Germany), quantified with a spectrophotometer (Perkin Elmer MBA2000, Fremont, CA), and then sequenced with an automated laser fluorescence sequencer (ABI PRISM 3100 Genetic Analyzer, Foster City, WI). Ten-fold serial dilutions of a known concentration of the product (from 100 pg/µl to 0.1 pg/µl) were used to establish the standard curve for real-time PCR. The real-time PCR conditions were as follows: 1 cycle for 20 seconds (sec) at 96°C, followed by 40 cycles of 2 sec at 96°C for denaturation, 15 sec at 60°C for annealing, and 15 sec at 72°C for extension. The melting program was performed at 72–95°C with a heating rate of 1°C per 45 sec. Spectral data were captured and analyzed using Rotor-Gene Real-Time Analysis Software 6.0 Build 14 (Corbett Research, Mortlake, Australia). All samples were run in triplicate. *Glyceraldehyde-3-phosphate dehydrogenase (GAPDH)* was analyzed as an endogenous RNA reference gene and gene expression was normalized to the expression of *GAPDH*.

### 5. Statistical analysis

To normalize the highly skewed distribution of mRNA expression levels of *C16orf74*, the data were natural log transformed and then back transformed for the interpretation of the results [34]. Receiver operating characteristics (ROC) curves were used to determine the optimal cutoff point of the mRNA level that yielded the highest combined sensitivity and specificity for progression. Using these values, patients were classified into high or low *C16orf74* expression groups. The Kaplan-Meier method was used to estimate time to progression, and differences were assessed using log-rank statistics. The prognostic value of *C16orf74* in terms of progression was analyzed using multivariate Cox proportional hazard regression models. Statistical analysis was performed using SPSS 12.0 software (SPSS Inc., Chicago, IL), and a p value of <0.05 was considered statistically significant.
